# Multiscale investigation of collagen structure in human skin and gel matrices using polarization resolved second harmonic generation microscopy

**DOI:** 10.1038/s41598-025-02536-4

**Published:** 2025-06-06

**Authors:** Mengyao Zhou, Madalena Pinto Gomes, Anouk Elgersma, H. Ibrahim Korkmaz, Bouke K. H. L. Boekema, Marie Louise Groot

**Affiliations:** 1 Faculty of Science, Department of Physics, Laserlab, Vrije Universiteit Amsterdam, De Boelelaan 1105, 1081HV Amsterdam, The Netherlands; 2 Department of Plastic Reconstructive and Hand Surgery, Amsterdam Movement Sciences (AMS) Institute, Amsterdam UMC, location VUmc, De Boelelaan 1117, 1081 HV Amsterdam, The Netherlands; 3 Alliance of Dutch Burn Care, Burn Research Lab, 1941 AJ Beverwijk, The Netherlands; 4 Department of Pathology, Amsterdam UMC, location AMC, Amsterdam, The Netherlands; 5 Department of Molecular Cell Biology and Immunology, Amsterdam Infection and Immunity (AII) Institute, Amsterdam University Medical Center (UMC), Location VUmc, Amsterdam, The Netherlands; 6 Burn Center and Department of Plastic and Reconstructive Surgery, Red Cross Hospital, Beverwijk, The Netherlands

**Keywords:** Collagen gel, Collagen type I and type III, Polarization second harmonic generation, Scar skin, Biophysics, Optics and photonics

## Abstract

Collagen is critical to the structure and function of skin tissues, with the collagen I/III ratios influencing fibrillogenesis, fiber organization, and skin mechanics. Abnormal collagen organization, such as in fibrosis or scar tissue, compromises both skin functionality and aesthetics. In this study, we employed label-free polarization resolved second harmonic generation (PSHG) microscopy to investigate collagen structure in artificial collagen matrices with various Col I/III ratios at the fibril scale ($\sim$1 to $3\,\upmu \hbox {m}$) and in ex vivo human healthy and scarred skin at the fiber scale ($\sim 10$ to $20\,\upmu \hbox {m}$). Complementary third harmonic generation (THG) microscopy provided additional structural information. Our results indicate that an increasing Col I/III ratio is associated with longer fibril length, higher PSHG intensity, and a reduced effective $\alpha$-helix pitch angle of fibrils. In pure Col I, the effective $\alpha$-helix pitch angle is determined to be $47.72^{\circ }$. These observations indicate alterations in fibril assembly. Furthermore, although the $\alpha$-helix pitch angle of fibers in both healthy and scarred skin was approximately $46.7^{\circ }$, healthy skin exhibited $24\%$ greater variability in fiber orientation, suggesting a more randomized organization compared to scar tissue. THG imaging further revealed a higher cellular density in scar tissue, consistent with the inflammatory activity associated with wound healing. Immunohistochemical (IHC) staining using dermatansulphate and Col III-specific antibodies confirmed that the Col I/III ratio is higher in healthy skin (2.2) than in scarred skin (1.6). These findings underscore the potential of PSHG microscopy for label-free, quantitative assessment of collagen structure across multiple scales, with THG offering complementary cellular insights. This integrated approach represents a promising strategy for real-time, in vivo monitoring and automated quantification of collagen organization in clinical applications, including dermatology, burn treatment, and fibrosis monitoring.

## Introduction

The extracellular matrix (ECM) is a dynamic, fibrillar network comprised of proteoglycans, fibrous proteins (e.g., fibronectins, collagens, elastins, fibrillins, and laminins), and the basement membrane^1^. These components not only maintain mechanical stability but also mediate biochemical signaling essential for tissue growth and repair. Collagen, the most abundant ECM protein, plays a critical role in various physiological and pathological processes, including breast cancer^2,3^, corneal diseases^4^, osteoarthritis^5^, wound healing^6^, and cardiovascular disorders^7^. In addition to stabilizing growth factors, regulating cell adhesion and signaling between cells and ECM, collagens are also employed in wound therapy to promote healing^6^.

During wound healing, remodeling processes alter the levels of collagen I (Col I) and collagen III (Col III), with the Col I/III ratio serving as a key indicator of ECM quality^8–10^. A favorable ECM quality during wound healing is characterized by an initial increase in collagen III during the early stages, followed by a restoration of the normal collagen I/III ratio as the tissue matures^11^. Mature Col I is key to mechanical stability, while Col III, associated with the early healing phase, produces thinner, more pliable fibers^12^. This balance regulates fibrillogenesis, the final fibril diameter and bundle structure, determining skin mechanics^13,14^. Disruption of this balance underlies pathological conditions, such as cardiac fibrosis^15–17^, which compromises heart function, and poor scar formation, which affects skin aesthetics and strength.

Conventional methods like immunohistochemistry (IHC) and western blot^18,19^ are widely used to analyze collagen isoforms but are invasive, labor-intensive, and often lack quantitative precision. These limitations underscore the need for a label-free, non-invasive imaging approach to assess collagen composition and fiber organization in situ.

Second harmonic generation (SHG) microscopy has gained recognition as a powerful label-free technique for evaluating fibrous tissues^20–23^. This nonlinear optical process occurs when non-centrosymmetric materials, such as collagen, generate light at twice the frequency (half the wavelength) of the incident light under intense pulsed illumination. Polarization second harmonic generation (PSHG) microscopy extends this approach by applying the dependency of SHG intensity on collagen fibril orientation relative to the incident light’s polarization. This polarization-sensitive technique enables precise quantification of fibril orientation, the second order susceptibility tensor and the average organization of the harmonophores, related to the alpha-helix pitch angle^24,25^. Campagnola’s group demonstrated SHG’s potential to differentiate collagen isoforms, such as Col I and Col V, in invasive breast carcinoma^26^. Increasing Col V content produced shorter, randomly distributed fibers with lower SHG intensity and reduced forward-backward (F/B) ratios. They further examined the ability of PSHG to distinguish Col I from Col III, revealing a decrease in SHG intensity and F/B ratios with higher Col III content^27^. These findings suggest that PSHG could emerge as a label-free technique to probe the ratio of specific collagen isoforms. Moreover, combined with Third harmonic generation (THG) in a single instrument, complementary information about tissue structure and cellular function can be resolved, enhancing our understanding of biological specimens^28,29^. THG is a nonlinear coherent process where three photons are upconverted to produce a photon that is triple the frequency of the incident photon. It is sensitive to local variations in third-order nonlinear susceptibility, refractive index, and dispersion and is particularly useful for visualizing tissue morphology at interfaces, as it provides strong contrast at optical discontinuities, such as lipid-rich components and cell membranes^30^.

In this study, we utilized PSHG microscopy to investigate the impact of collagen composition on fibril and fiber organization in artificial collagen matrices with various Col I/III ratios and in human skin, while including THG where it aids in contextualizing tissue structure. An automated pixel-based method was developed to quantify collagen fiber orientation, second-order susceptibility ratios and the alpha-helix pitch angle, providing a comprehensive framework for characterizing collagen properties at both fibril and fiber scales. Parameters such as SHG intensity, fibril length, thickness, second-order susceptibility ratios and the alpha-helix pitch angle were analyzed to identify compositional differences. Additionally, collagen fibers from both healthy and scarred skin were analyzed using PSHG microscopy and IHC staining to compare with the fibril characteristics.

## Materials and methods

### Sample preparation

Multiple types of samples were used in this study, including human skin tissues (both fresh and histological sections), artificial collagen matrices, mouse skin, and starch granules.

#### Human skin tissue samples

Skin tissue samples were obtained from adult patients that underwent elective surgery at the Departments of Surgery or Plastic and Reconstructive Surgery of the Red Cross Hospital, Beverwijk. Scar skin specimens from 7 different donors were collected (Average donor age: 34.5; sex: $67\%$ female, average scar age: 7.4 year). Healthy skin samples were collected from 5 different donors. Sample collection was based on availability while ensuring a diverse representation of skin types. To maintain consistency in collagen analysis, we included only samples with well-defined healthy and scarred skin regions, excluding cases with mixed tissue types or active infections that could introduce variability. Oral informed consent was obtained from all subjects, and surgical waste tissue was used for scientific purposes when patients had not objected, and study was performed in accordance with national and relevant guidelines (https://www.coreon.org/, accessed on 23 November 2020; The Act on the Medical Treatment Agreement, Article 7:467 BW). Consent for the use of these anonymized, post- operative residual tissue samples was received through the informed opt-out protocol of the Red Cross Hospital. All procedures on human tissue were performed under national guidelines (https://www.coreon.org/accessed on 23 November 2020) and with approved by the institutional privacy officers.

After collecting skin samples in vivo, the sample were fixed by formaldehyde solution ($4\%$ or kryofix ($50\%$ ethanol $96\%, 43\%$ ddH20, $7\%$ PEG 300) and processed for sectioning according to standard methods. For histological analysis, tissue sections ($5\,\upmu \hbox {m}$) were stained with hematoxylin/eosin (H&E). For immunohistochemical analysis, sections for Col III staining were pretreated with 10 mM citrate buffer (pH 6) at $70^{\circ }\hbox {C}$ for 10 min followed by 20 min cool-down in PBS. Sections were incubated with primary antibody (Abcam AB6310) overnight at $4^{\circ }\hbox {C}$ (dilution 1:200) followed by incubation with a poly-HRP-goat-anti-mouse secondary antibody (BrightVision, Immunologic) for 30 min at room temperature. For dermatansulphate (DS), sections were incubated with antibody GD3 A12 (courtesy of W. Daamen, Radboudumc, Nijmegen, dilution 1:20) at room temperature for 1 h, followed by incubation with mouse anti-VSV antibody P5D4 (courtesy of W. Daamen, Radboudumc, Nijmegen, dilution 1:10) at room temperature for 1 h and incubation with a poly-HRP-goat-anti-mouse secondary antibody (BrightVision) for 30 min at room temperature. Peroxidase activity was detected with $3,3'$-diaminobenzidine substrate. All sections were counterstained with hematoxylin, dehydrated and mounted with Eukitt Mounting Medium (Merck).

In addition to histological sections, experiments were also conducted on fresh human skin samples. The fresh human skin samples were cut into $1 \times 0.5 \times 0.8\,\hbox {mm}^3$ sections, and placed with the cross-section facing the objective for imaging.

#### Artificial collagen matrices

Artificial collagen matrices with Col I/III ratios of 1:0, 9:1, 4:1, 7:3, 3:2 and 0:1 were prepared by mixing human type I collagen solution ($2.9 \sim 3.2$ mg/ml, catalog No. 5007, Advanced Biomatrix, Carlsbad, CA) and human type III collagen solution ($\sim 1$ mg/ml, catalog No. 5021, Advanced Biomatrix, Carlsbad, CA). The mixtures were then neutralized to a pH of approximately 7 using a preformulated neutralizing solution (Catalog No. 5155, Lot No. 9111, Advanced Biomatrix, Carlsbad, CA) and diluted with $10\times$ phosphate-buffered saline (PBS) to achieve a final gel concentration of 1.58 mg/ml in a total volume of 1.268 ml. The detailed amounts of the components are provided in Table S1. The collagen gel solutions were polymerized overnight at $37^{\circ }\hbox {C}$ in six separate petri dishes. Comparisons of the PSHG signal from the different compositions were conducted using gels polymerized simultaneously, ensuring that the results for each concentration were internally consistent.

#### Mouse skin samples

The mouse skin was excised from leftover wild type female mice (Euthanasia was performed under isoflurane anesthesia, all efforts were made to minimize suffering), provided by Neuroscience Amsterdam (CNCR, VU Amsterdam). We removed the hair from dorsal skin region using scissor and a chemical depilatory agent (Veet, Reckitt Benckiser, Canada). Then we excised the skin to obtain $10 \times 10\,\hbox {mm}^2$ skin samples. All experimental procedures on mouse skin tissues were approved by the Animal Ethics Committee of VU University and carried out in accordance with the European Council Directive (2010/63/EU) by permission of the Animal Research Law of the Netherlands. All procedures were conducted following the ARRIVE 2.0 guidelines on animal research.

#### Starch granule

Starch granule used for calibration of PSHG microscope setup were bought from supermarket (MAIZENA, Albert Heijn). Samples were prepared by dissolving 0.05 g starch in 3 ml water.

### Polarization second harmonic generation microscope setup

**Fig. 1:**
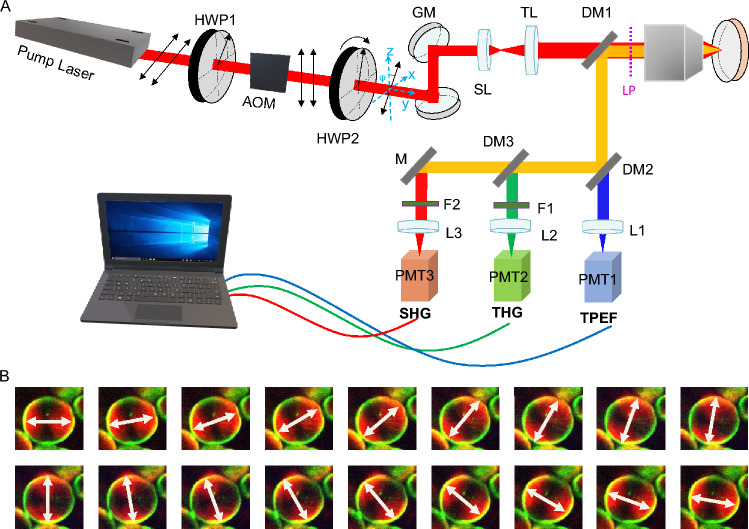
(**A**) Schematic diagram of the experimental setup. SL, scan lens; TL, tube lens; M, mirror; L1, L2, L3 focusing lens. (**B**) Starch granule images with 18 polarizations (from $0^{\circ }- 170^{\circ }$ in step size of $10^{\circ }$). White arrows indicate the polarization angles of the laser.

Figure 1A shows our experimental setup. We used a fiber laser with a pulse duration of less than 50 fs and a central wavelength of 1050 nm (Tidal, Valo Innovations). An acousto-optic modulator (AOM, MT250-A0.5–1064, Opto Electronic) was used as pulse picker to reduce the repetition rate of the laser from 30 to 1 MHz, to keep the average power low (around 5 mW), but the peak power high enough to excite nonlinear signals. One half-wave plate (HWP1) was used to rotate the polarization state of the laser from horizontal to vertical to match the requirement of the AOM. A second half-wave plate (HWP2) was placed before the galvo scanner mirrors (GM) with a piezoelectric rotating frame to quickly switch the polarization angle of the laser. The laser was focused onto the sample by a $40 \times /1.3$ oil-immersion objective (Nikon S Fluor, Nikon), resulting in a focus of size $0.4 \times 0.4 \times 2\,\upmu \hbox {m}^3$. The imaging depth was $10\,\upmu \hbox {m}$ for fresh samples and $2\upmu \hbox {m}$ for thin frozen sections. The generated signals were detected in epi-direction, filtered from the 1050 nm fundamental photons by a dichroic mirror (DM1, FF872-Di01, Semrock), and subsequently divided into three channels through two other dichroic mirrors (DM2, LP580; DM3, LP442; Semrock). Two bandpass filters (F2, FF01-520/35-25; F3, FF01-355/40-25; Semrock) were placed in the detection arm of the SHG and third harmonic generation (THG). Three photomultiplier tubes (PMT1, H10721-20; PMT2, H16201-40; PMT3, H10721-210; Hamamatsu, Japan) were used to detect the signals. In this report we will mainly show the SHG images while including THG where it aids in contextualizing tissue structure. Microscopy data were recorded using LabView software (Flash Pathology B.V., Amsterdam, The Netherlands).

To assess the depolarization effect of the optical elements between HWP2 and the objectives, we first introduced a linear polarizer (LP) just before the objective lens and recorded the LP angle corresponding to the maximum transmitted power through the objective, which is approximately twice the rotation angle of HWP2. Then we removed the LP, and through rotation of HWP2, a series of 18 images of starch granule was captured at polarization angles ranging from $0^{\circ }$ to $170^{\circ }$ with a $10^{\circ }$ interval (See Visualization 1.). As depicted in Fig. 1B, the orientations of maximum intensity of SHG aligned closely with the polarization angle of the incident laser. Consequently, we infer that the depolarization effect in our light path is negligible, affirming the readiness of the setup for further experimentation.

### Western blot

Protein expression was evaluated through immunoblotting. Briefly, samples were lysed in denaturing Laemmli buffer (1:3). For each sample, an aliquot of $25\,\upmu$l was boiled at $95^{\circ }$C for 5 min and loaded in a Bolt $4-12\%$ Bis-Tris Plus Wedge Well gel (Invitrogen, Carlsbad, USA). Proteins were separated using SDS-PAGE and transferred. Following transfer onto PVDF membrane. The membranes were blocked with $5\%$ milk in Tris-Buffered Saline with $0.1\%$Tween 20 (TBST) for 1 h at room temperature (RT). The membranes were then incubated with collagen type anti-Col I antibody (1:2000, PAB10190, Abnova) in $5\%$ milk in TBST overnight at $4^{\circ }\hbox {C}$ and washed three times with TBST for 10 min each wash. The membranes were then incubated with secondary antibody (1:2500, P0448, Dako) in $5\%$ milk in TBST for 1 h at RT, and again washed five times with TBST for 10 min each wash. Visualization was performed using Pierce pico-ECL Western kit reagent (Thermo Scientific, Rockford, USA), and the membranes were developed using Amersham ImageQuant 800.

### Data analysis

The PSHG images were preprocessed with normalization and segmentation with threshold of 0.2 to remove the background. This threshold was chosen manually based on empirical testing across multiple samples to optimize the balance between structure inclusion and background exclusion, ensuring that only meaningful signal contributions were analyzed.

According to^31^, the intensity of the PSHG response can be fitted by the following expression:

1 $$\begin{aligned} I_{SHG}(\phi ) = C^2sin^22(\phi -\alpha )+[Asin^2(\phi -\alpha )+Bcos^2(\phi -\alpha )]^2 \end{aligned}$$

where $\phi$ is the polarization angle of incident beam, $\alpha$ represents the orientation of the fibers. $A=I_0\chi _{31}, B=I_0\chi _{33}$ and $C=I_0\chi _{15}, I_0$ is proportional to the intensity of the incident beam, and $\chi _{31}, \chi _{33}$ and $\chi _{15}$ are the non-zero elements of the second order susceptibility tensor characterizing the tissue under cylindrical symmetry assumption^32^. It is worth noting that transforming Eq. (1) into a Fourier series can accelerate the fitting procedure allowing for the retrieval of all parameters from Eq. (1) by Eq. (2):

2 $$\begin{aligned} I_{SHG}(\phi ) = a_0 +a_2cos2\phi +b_2sin2\phi +a_4cos4\phi +b_4sin4\phi \end{aligned}$$

where

3 $$\begin{aligned} \left\{ \begin{array}{lr} a_0 = \frac{1}{T}\int _{-\frac{T}{2}}^{\frac{T}{2}}I_{SHG}(\alpha )d\alpha = Mean (I_{SHG}(\alpha )), & \\ a_n = \frac{2}{T}\int _{-\frac{T}{2}}^{\frac{T}{2}}cos(n\alpha )I_{SHG}(\alpha )d\alpha = 2*Mean(cos(n\alpha )I_{SHG}(\alpha )) , & \\ b_n = \frac{2}{T}\int _{-\frac{T}{2}}^{\frac{T}{2}}sin(n\alpha )I_{SHG}(\alpha )d\alpha = 2*Mean(sins(n\alpha )I_{SHG}(\alpha )). & \end{array} \right. \end{aligned}$$

Furthermore, according to^33^, the molecular origins of the second-order susceptibility involve both peptide and methylene groups. The average organization of the harmonophores, related to the $\alpha$-helix pitch angle ($\theta ^p$) for the peptide groups, can be determined by:

4 $$\begin{aligned} tan^2\theta ^p = \frac{2}{\chi _{33}/\chi _{31}-\chi _{15}/\chi _{31}+1} \end{aligned}$$

With these calculations as a foundation, we constructed an automated image analysis framework utilizing MATLAB. At the pixel level, we analyzed the angle ($\alpha$) of the fibers, along with the ratios of second-order susceptibility ($\chi _{15}/\chi _{31}, \chi _{33}/\chi _{31}$), as well as the $\alpha$-helix pitch angle ($\theta ^p$).

Additionally, to quantify the intensity of PSHG and Western Blot bands, ImageJ software was utilized. For the analysis of fibril length and thickness, images obtained at the horizontal polarization state were processed in ImageJ, with measurements taken from five randomly selected regions of interest (ROIs) per sample. IHC images were quantified using brown color threshold in ImageJ.

### Statistical analysis

Statistical analysis was performed using Origin (Version OriginPro 2022b, Academic.). Groups were compared using analysis of variance (ANOVA). The significance threshold was set at p<0.05.

## Results

### PSHG distinguishes different structures

**Fig. 2:**
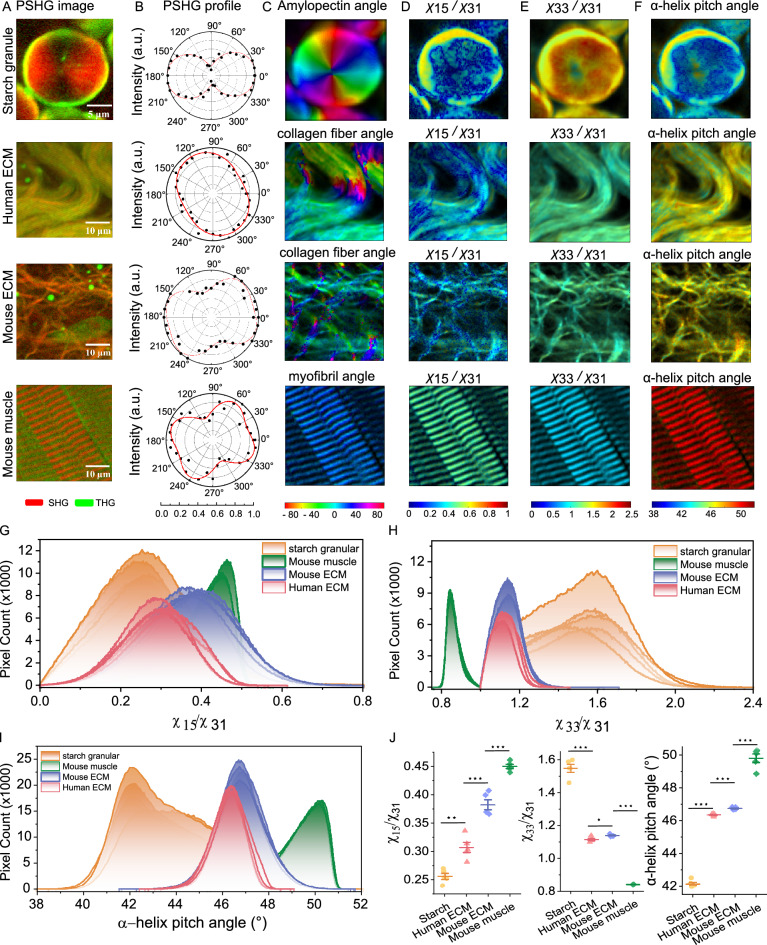
Polarization resolved SHG measurements on different structures. (**A**) PSHG images (red) recorded at polarization angle $\phi =0^{\circ }$ of starch granule, human and mouse ECM, mouse muscle, alongside THG signals (green). (**B**) PSHG intensity profiles of a randomly selected pixel of interest. Color maps (**C**-**F**), frequency distributions (**G**-**I**), and mode values (**J**) of structure angle, $\chi _{15}/\chi _{31}, \chi _{33}/\chi _{31}$, and $\alpha$-helix pitch angle for amylopectin, collagen fibers, and myofibrils. Same color represents same structure, one curve/dot represent one sample. * stands for p<0.05, ** for p<0.01, *** for p<0.001 and ns for no significant difference.

Within this section, we investigate the capability of PSHG imaging to differentiate various biological structures, including starch granules, human ECM, mouse ECM, and mouse muscle. The mouse muscle was observed within the mouse skin specimens analyzed in this study. Under the same laser power conditions, we captured SHG signals from amylopectin in starch granule, collagen fiber in human and mouse ECM, and myofibril in mouse muscle. The PSHG images recorded at polarization angle $\phi =0^\circ$ are shown in Fig. 2A (PSHG images for all 18 polarization angles are provided in Visualization 1–4.). Single-peaked and double-peaked PSHG intensity profiles were observed, as shown in Fig. 2B. A single peak in the PSHG intensity profile occurs in regions with uniform orientation and symmetry, where the polarization of the incident light aligns with the structural organization, enhancing second harmonic signal generation. In contrast, regions where $\chi _{33}/\chi _{31} =1$ (cyan regions in Fig. 2E, particularly in mouse muscle) exhibit double peaks in the PSHG intensity profile. This condition indicates that the two fundamental photons in either the z-direction or the x-direction can generate equal SHG intensities in z-direction, resulting in two peaks in PSHG profile. These peaks provide valuable insights into the alignment of light with second order susceptibilities and distinct structural orientations.

Color maps of the derived $\alpha , \chi _{15}/\chi _{31}, \chi _{33}/\chi _{31}$ and $\theta ^p$ are depicted in Fig. 2 C-F, with their corresponding frequency distributions displayed in Fig. 2 G-I based on measurements from five samples of each structure. As illustrated in Fig. 2J, the mode values of $\chi _{15}/\chi _{31}, \chi _{33}/\chi _{31}$ and $\theta ^p$ for starch granules, human ECM, mouse ECM, and mouse muscle show significant differences (p < 0.05). We observed a mode helix angle $\theta ^p$ of $42^\circ$ for starch granules, 46$^\circ$ for collagen, and $50^\circ$ for muscle (see Table S2). These results are compatible with previous studies that report amylopectin helix angles around $39^\circ$ using X-ray diffraction^34,35^, collagen filament angles around $45^\circ$^36,37^, and muscle myofibril in the range of $50-60^\circ$^32,38^. These findings suggest that PSHG microscopy can differentiate these distinct biological structures by analyzing their second-order susceptibility ratios and $\alpha$-helix pitch angles. Detailed mode values for these structures are summarized in Table S2.

### PSHG distinguishes Col I and Col III

**Fig. 3:**
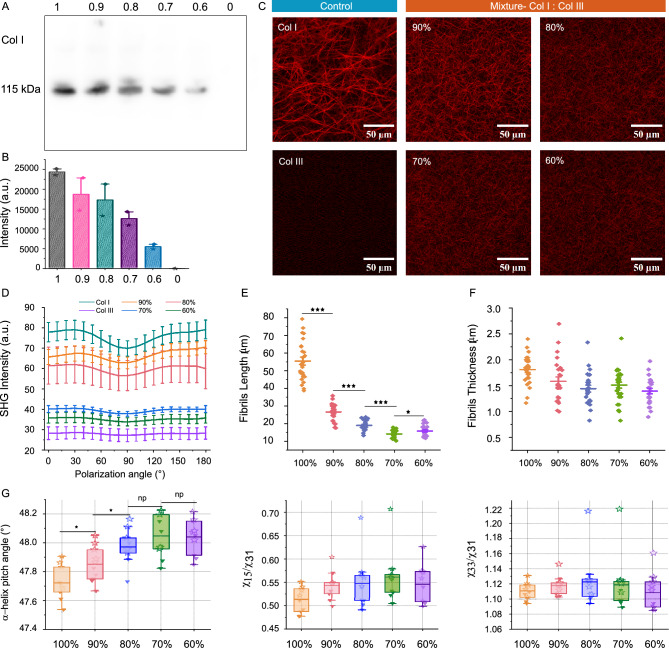
(**A**) Western blot (WB) images show bands around $\sim$115 kDa in collagen gel samples with decreasing Col I proportions, detected using a Col I antibody. Lane numbers correspond to the Col I proportions. A representative cropped blot is shown, with all original blots provided in Supplement 1, Figure S1. (**B**) Quantitative analysis showing the Col I incorporation (exposure time: 100 s). Data from two individual blots are represented in the graph. (**C**) PSHG images of collagen gel matrices recorded at polarization angle $\phi =0^\circ$. (**D**) Mean SHG intensity profiles with different polarization angles. Collagen fibrils (**E**) length and (**F**) thickness in matrices with different Col I/III ratios. (**G**) The mode values of $\chi _{15}/\chi _{31}, \chi _{33}/\chi _{31}$ and $\alpha$-helix pitch angle of different collagen gel matrices. Triangles represent the results of trial 1, and stars represent the results of trial 2. For each trial 5 ROIs were measured.

We further investigated the ability of PSHG imaging to discriminate among different ratios of Col I and III. Collagen gel solutions were prepared with Col I/III ratios of 100:0, 90:10, 80:20, 70:30, 60:40 and 0:100%. Col I content in gels were analyzed by Western blot. Figure 3A shows a cropped western blot bands captured at 100 s exposure times, with the numbers above the lanes indicate the proportion of the Col I in each sample (All original blots are presented in Supplement 1, Figure S1). The overlaid image showed that the blot intensities for 100% and 90% Col I were saturated at 150 and 200 s exposure times, indicating a high level of Col I presence (Fig. S1 A). Figure 3B presents a quantitative analysis of blot intensities, recorded with 100 s exposure time and derived from two individual western blot experiments. As the proportion of Col I decreased, a corresponding decrease in band intensity was observed, with the 0% Col I sample showing near-zero intensity. This trend confirms the expected reduction in Col I content. However, precise quantification of Col I proportions based solely on blot intensity proved challenging. Here, we use the Western blot results as a guideline to interpret the PSHG data.

The mixed collagen gel solutions were polymerized overnight at 37$^{\circ }\hbox {C}$ in petri dishes. SHG imaging indicated significant differences in the collagen matrices with various Col I/III ratios(Fig. 3C). In the control sample containing 100% Col III, no fiber formation was detected, and the SHG signal was primarily noise. SHG intensity was positively correlated with an increase in the proportion of Col I, with the mean SHG intensity of the 80:20% Col I/III samples being $\thicksim$ 1.7 times higher than that of the 60:40% Col I/III samples (Fig. 3D). The thickness of the collagen structure did not exhibit a clear trend, with mean value remaining within the range of 1.3 - 1.8 $\upmu$m (Fig. 3F), consistent with the normal fibril thickness^39^. As the proportion of Col I increased, there was a notable increase in fibrils length (Fig. 3E). Despite being based on only two experimental iterations, these findings suggest that the Col I/III ratio is a key determinant of collagen matrix structure. Both SHG intensity and collagen fibril length were significantly influenced by variations in the Col I/III ratio.

Next, the second order susceptibility ratios and $\alpha$-helix pitch angle were analyzed using our pixel-based polarization analysis program. Figure 3G presents the mode values of $\chi _{15}/\chi _{31}, \chi _{33}/\chi _{31}$ and $\alpha$-helix pitch angle for the collagen gel matrices. Collagen gel matrices with Col I/III ratios of 90:10, 80:20, 70:30, and 60:40 exhibited statistically significant differences in the $\alpha$-helix pitch angle compared to the control sample (100:0% Col I/III) at p < 0.05 level. The $\alpha$-helix pitch angle showed a clear increasing trend as the proportion of Col III increased, suggesting that Col III influences the collagen fibrils structure, potentially making them more relaxed or extended. The second order susceptibility ratios did not show a clear trend above the standard deviation. The extracted values for $\chi _{15}/\chi _{31}$ ranged from 0.51 to 0.62, $\chi _{33}/\chi _{31}$ ranged from 1.11 to 1.16, and $\theta ^p$ ranged from $47.60^{\circ }$ to $48.19^{\circ }$. A summary of the averaged mode values for these parameters is provided in Table S3.

### Normal skin and scar skin

To further investigate the potential of the PSHG in probing the ratio of Col I and III in real skin tissue, we conducted PSHG measurements on both healthy skin (n=5) and scar skin tissue (n=7).

**Fig. 4:**
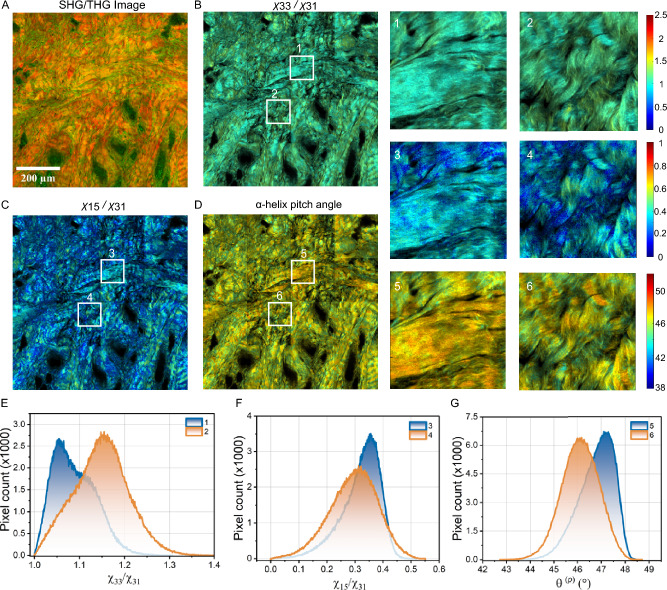
(**A**) SHG/THG images of cross section of a fresh hypertrophic scar skin sample, measured with the cross-section face to the objective. Color maps (**B** - **D**) and histogram distributions (**E** - **G**) of $\chi _{33}/\chi _{31}, \chi _{15}/\chi _{31}$ and $\alpha$-helix pitch angle for two boxed regions.

Figure 4A displays the SHG/THG image of the dermis layer from a fresh hypertrophic scar sample, captured with the cross-section facing the objective. Figure 4B - D respectively present the color maps of $\chi _{33}/\chi _{31}, \chi _{15}/\chi _{31}$, and the $\alpha$-helix pitch angle, with neighbor pixel averaging. Two regions are magnified for detailed analysis, with corresponding tensor ratio images and $\alpha$-helix pitch angle images labeled 1–6. These regions include one where fibers are well-aligned and another where fibers are more randomly oriented and curved. Fig. 4E-G depict the histogram distributions of $\chi _{33}/\chi _{31}, \chi _{15}/\chi _{31}$ and the helix pitch angle for the two selected regions. The histogram of $\chi _{33}/\chi _{31}$ shows distinct peaks at 1.05 for region 1 and 1.15 for region 2. The $\chi _{33}/\chi _{31}$ ratio in region 1, being closer to 1, indicates higher molecular order and alignment, suggesting a more structured and less anisotropic collagen matrix. Conversely, region 2, with a higher $\chi _{33}/\chi _{31}$ value, reflects greater molecular disorder. Similarly, the mode values of $\chi _{15}/\chi _{31}$ of the regions 3 and 4 were 0.35 and 0.31, respectively, further illustrates differences in the anisotropic properties of the collagen fibers. The $\alpha$-helix pitch angles for regions 5 and 6 were $47.1^{\circ }$ and $46.1^{\circ }$, respectively, revealing subtle variations in the collagen’s helical structure. These three parameters ($\chi _{33}/\chi _{31}, \chi _{15}/\chi _{31}$, and $\alpha$-helix pitch angle) provide high resolution insights into local collagen fiber arrangement and molecular order, making them potentially valuable tools for quantifying collagen tissue structure.

**Fig. 5:**
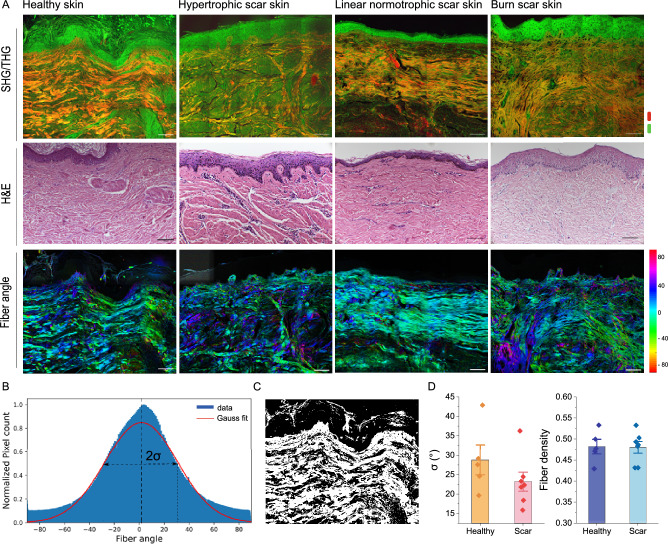
(**A**) SHG/THG, Hematoxylin and eosin (H&E) staining images, and fiber orientation maps of frozen sections from healthy skin and three types of scar tissue. Scale bar 100 $\upmu$m. Total area is $1 \times 0.8\,\hbox {mm}^2$. (**B**) Fiber orientation distribution and (**C**) fiber mask. (**D**) Quantitative analysis of the standard deviation of fiber orientation distribution and fiber density of healthy skin (n=5) and scar skin (n=7).

**Fig. 6:**
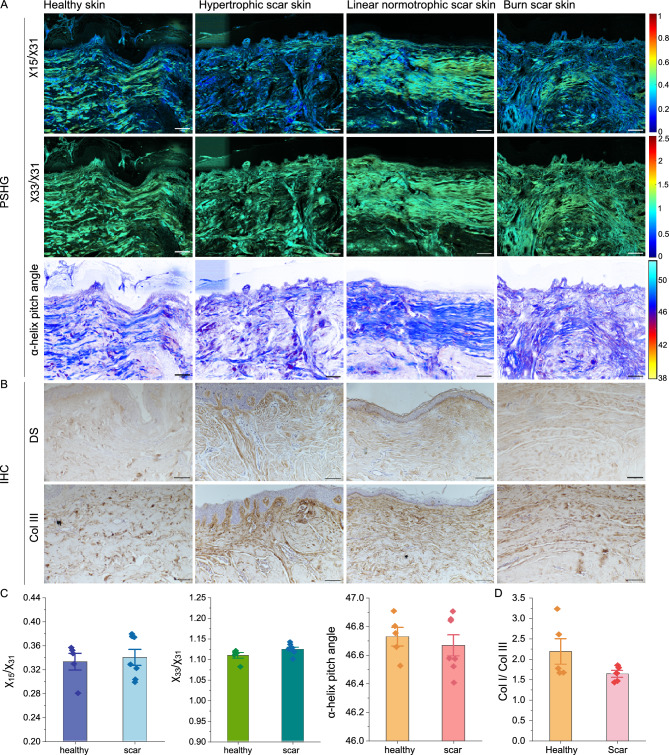
PSHG and IHC analysis of healthy and scar skin tissue. (**A**) Color maps of $\chi _{15}/\chi _{31}$, $\chi _{33}/\chi _{31}$ and $\alpha$-helix pitch angle, providing collagen structure information. (**B**) IHC staining for dermatansulphate and Col III. Scale bar 100 $\upmu$m. (**C**) Mean mode values of $\chi _{15}/\chi _{31}$, $\chi _{33}/\chi _{31}$ and $\alpha$-helix pitch angle of healthy (n = 5) and scarred (n = 7) skin samples. (**D**) The ratio of Col I/III analyzed from IHC images. Dermatansulphate was used as proxy for Col I. Shown in mean, error bars represent SD.

To validate the ability of PSHG in characterizing scar tissue, we conducted a direct one-to-one comparison between PSHG analysis and standard histological analysis using frozen tissue sections.

Figure 5A presents the SHG/THG, H&E stained images, and collagen fiber orientation maps of representative cross-sections of healthy skin and three types of scar tissue. The SHG/THG and H&E images show higher cellular density in all three types of scar tissue compared to healthy skin, consistent with the inflammatory activity associated with wound healing. Healthy skin samples displayed a broader range of colors in the fiber orientation map, indicative of a randomized fiber arrangement. Conversely, scar tissues showed less color variation, suggesting more aligned fiber structures. To quantify fiber orientation, the fiber orientation distribution was fitted to a Gauss function, and the standard deviation was extracted (Fig. 5B). Healthy skin exhibited a 24% higher standard deviation compared to scarred skin, confirming more random fiber arrangement (Fig. 5D). Additionally, Fig. 5C shows a binary mask of collagen fibers generated using a threshold of 0.45 times the mean intensity. The analysis shows that no significant difference in collagen fiber density was observed between healthy and scarred skin tissue (Fig. 5D).

Figure 6 displays the PSHG analysis images and IHC images of representative cross-sections of healthy skin and three types of scar tissue. In Figure 6A, the color maps of $\chi _{15}/\chi _{31}$, $\chi _{33}/\chi _{31}$ and $\alpha$-helix pitch angle reveal subtle differences between healthy and scarred skin. The mean mode values of $\chi _{15}/\chi _{31}, \chi _{33}/\chi _{31}$ and $\alpha$-helix pitch angle were approximately $0.3, 1.1, 46.7^{\circ }$, respectively, for both healthy and scarred skin (Fig. 6C), with detailed values provided in Table S4. Figure 6B presents the corresponding IHC images, showing the distribution of dermatansulphate (as a proxy for Col I) and Col III proteins in the same tissue samples. Quantification of the IHC results is shown in Fig. 6D. The Col I/III ratio averaged approximately 2.2 in healthy skin and 1.6 in scarred skin. This reduction in the Col I/III ratio for scar tissue indicates a relative increase in Col III, consistent with its known role in wound healing and scar formation.

## Discussion

PSHG microscopy is a promising imaging modality that not only provides high-quality imaging but also enables quantitative analysis of collagen organization. This is facilitated by the second-order susceptibility tensor, which comprises 27 matrix elements that allow for the extraction of intricate molecular properties associated with collagen and other fibrous proteins.

Although PSHG imaging can be influenced by artifacts, such as tissue scattering and intrinsic birefringence, we minimized these influences by restricting our measurements to superficial regions in fresh tissues ($\sim 10 \upmu \hbox {m}$ depth) and in thin frozen sections ($\sim$ 2um depth, total sample thickness 5 um), with signal collected in the back scatter direction^40,41^. This configuration minimizes distortions and ensures reliable data acquisition. However, for deeper tissue imaging, phase retardation $\triangle \phi$ induced by birefringence must be considered. Future studies should incorporate advanced models that account for birefringence effects in PSHG, such as reported by Yang et al.^41^. Additionally, to maintain consistency and minimize variability due to depth-dependent artifacts, we conducted PSHG analysis within the same sample group-either fresh tissues or frozen sections-ensuring a uniform imaging depth for reliable comparisons.

Analysis of fiber orientation and distribution by PSHG show that healthy skin exhibits a 24% higher standard deviation in fiber orientation, i.e. a more random fiber organization, than in scarred skin. This finding aligns with previous histopathological studies by Verhaegen et al and Van Zuijlen et al^42,43^, which reported healthy skin contained significantly thicker collagen bundles with greater spacing between them, whereas scar tissue and connective tissue disorders displays a more parallel and aligned collagen bundle arrangement^43^. In contrast to these previous methods, our approach not only yields collagen fiber orientation but also reveals the local supramolecular alpha-helix pitch angle - a structural feature linked to the orientation of C-N peptide bonds^24^. This additional insight provides a significant potential advantage for detailed tissue analysis and could advance diagnostic capabilities. For example, Tokarz et al. used PSHG to characterize pathological thyroid tissue in four carcinoma types^44^. More recently, Chiara Stringari et al. experimentally characterized PSHG signals in live zebrafish larvae, demonstrating the ability of PSHG to differentiate between collagen, myosin, and tubulin structures in intact tissue samples^24^. They also introduced a numerical model that can accurately reproduce PSHG measurements obtained from collagen, myosin, microtubule, and actin structures, revealing the precise dependence of SHG signals on the 3D distribution of the peptide bonds within protein assemblies.

To compare PSHG-derived collagen parameters directly one-on-one with immunohistochemistry (IHC), we performed our analysis on frozen tissue sections. However, it is important to note that tissue fixation can induce collagen cross-linking and shrinkage, potentially affecting SHG intensity and anisotropy measurements. Due to practical reasons, it was hard to locate exactly the same regions for PSHG and IHC. Instead, we analyzed an area of $1 \times 0.8\,\hbox {mm}^2$ per patient for both PSHG and IHC and grouped the samples into healthy and scar skin groups. Further studies comparing fresh and fixed tissues will be necessary to fully evaluate the impact of fixation on PSHG measurements. Additionally, we selected dermatan sulfate (DS) staining instead of conventional Col I antibodies for IHC, as DS is more sensitive to detect cell-synthesized collagen and provides a more dynamic representation of collagen organization in both in vitro and in vivo models^45^.

To investigate how Col I/III ratios influence collagen morphology, we engineered self-assembled collagen gel matrices with controlled Col I/III ratios (100:0, 90:10, 80:20, 70:30, 60:40, and 0:100%). All collagen gels were self-assembled simultaneously to ensure comparability. The observation that increased Col III content in collagen gel matrices results in lower SHG intensity is consistent with that made by Campagnola et al.^27^, who suggested that the Col I and III isoforms could be randomly combined within the same fibrils. Furthermore, we observed that changes in Col I/III ratios impact fibril length, anisotropy ($\chi _{15}/\chi _{31}, \chi _{33}/\chi _{31}$), and $\alpha$ helix pitch angle, suggesting that PSHG has potential as a label-free tool for assessing ECM integrity and remodeling. The histogram distributions of the $\chi _{15}/\chi _{31}, \chi _{33}/\chi _{31}$ and $\alpha$ helix pitch angle in two distinct regions of a fresh hypertrophic scar sample (Fig. 4) are significantly different, indicating a large heterogeneity within the tissue. However, our comparison of healthy (n = 5) and scarred skin (n = 7) samples did not reveal significant differences in these PSHG parameters, likely due to the inherent complexity of real tissue compared to the controlled gel matrix system and the sensitivity of PSHG-derived parameters to local variations in collagen organization. This highlights the need for further research to establish robust correlations between PSHG-derived parameters, histopathological grading, and patient outcomes. Defining clinically relevant diagnostic thresholds will be crucial for translating PSHG into real-world applications, including dermatology, burn treatment, and fibrosis monitoring.

Furthermore, PSHG can image and quantify the collagen fiber organization and substructural features in fresh, frozen or deparaffinized tissue sections, which could be added to the existing histology without altering customary hospital sample preparation. Additionally, PSHG can be integrated into an epi-detection setup within an endoscopic system, enabling its application in clinical settings^46^. Notably, Radaelli et al. have successfully employed SHG-based ECM analysis in living, anesthetized animal models, demonstrating its feasibility for in vivo imaging^47^.Future studies should focus on adapting PSHG for time-lapse imaging of wound healing and fibrosis progression in preclinical models, followed by validation in human subjects. Such efforts will be crucial for assessing PSHG’s clinical utility and establishing its role in precision diagnostics for collagen-related pathologies.

## Conclusion

This study highlights the potential of PSHG microscopy as a label-free tool for quantifying collagen organization at both fibril and fiber scales. By analyzing collagen matrices with varying Col I/III ratios and ex vivo human skin samples, we demonstrated that PSHG-derived parameters-such as $\chi _{15}/\chi _{31}, \chi _{33}/\chi _{31}$ and $\alpha$ helix pitch angle-reflect structural changes in collagen organization. Additionally, the integration of THG imaging provided complementary cellular information. While our findings support the utility of PSHG in assessing extracellular matrix integrity, further studies are needed to refine depth-resolved imaging models and establish clinically relevant diagnostic thresholds. With its capability for real-time, label-free imaging, PSHG has strong potential for clinical applications in dermatology, wound healing, and fibrosis monitoring.

## Supplementary Information

Supplementary Information 1.

Supplementary Information 2.

Supplementary Information 3.

Supplementary Information 4.

Supplementary Information 5.

## Data Availability

The data that support the findings of this study are available from the corresponding author upon reasonable request.
