# Supplementary material for: Multiscale investigation of collagen structure in human skin and gel matrices using polarization resolved second harmonic generation microscopy
Source: Sci Rep. 2025 Jun 6;15:20025. doi: 10.1038/s41598-025-02536-4 (PMC12144187; doi:10.1038/s41598-025-02536-4)
Supplement: Supplementary file 1 — Supplementary Information 1. [file 41598_2025_2536_MOESM1_ESM.pdf]

Supplementary information for "Multiscale investigation of collagen structure in human skin and gel matrices using polarization resolved second harmonic generation microscopy"

Mengyao Zhou[1,*], Madalena Pinto Gomes[2,3,4], Anouk Elgersma[3], H.Ibrahim Korkmaz[2,4,5,6], Bouke K.H.L. Boekema[2,3], and Marie Louise Groot[1]

[1]Faculty of Science, Department of Physics, Laserlab, Vrije Universiteit Amsterdam, De Boelelaan 1105, 1081HV, Amsterdam, The Netherlands
[2]Department of Plastic Reconstructive and Hand Surgery, Amsterdam Movement Sciences (AMS) Institute, Amsterdam UMC location VUmc, De Boelelaan 1117, Amsterdam, 1081 HV, The Netherlands
[3]Alliance of Dutch Burn Care, Burn Research Lab, Beverwijk, 1941 AJ, The Netherlands
[4]Department of Pathology, Amsterdam UMC, location AMC, Amsterdam, The Netherlands
[5]Department of Molecular Cell Biology and Immunology, Amsterdam Infection and Immunity (AII) Institute, Amsterdam University Medical Center (UMC), Location VUmc, Amsterdam, Netherlands
[6]Burn Center and Department of Plastic and Reconstructive Surgery, Red Cross Hospital, Beverwijk, The Netherlands
[*]m.zhou@vu.nl

Supplementary Figure

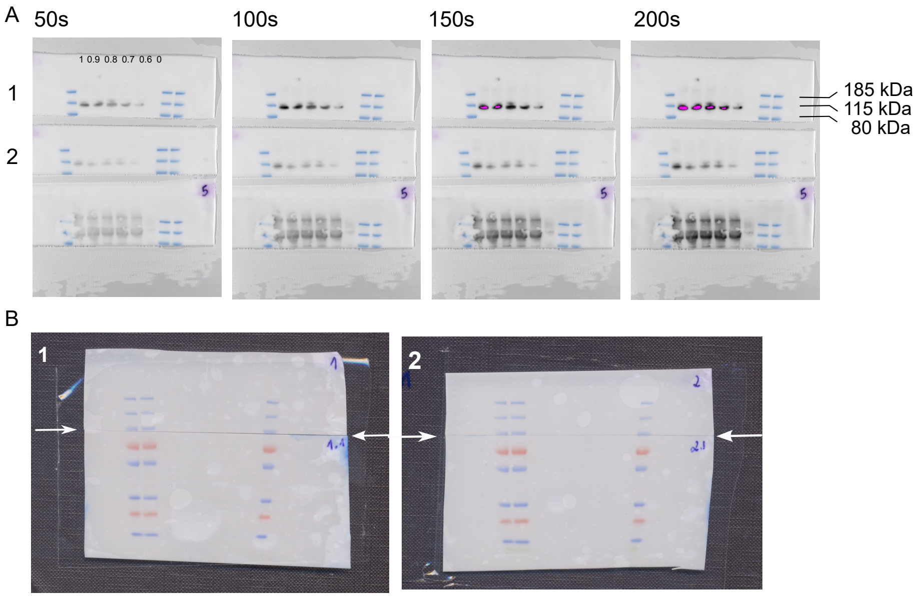

Figure S1. (A) Original blots with membrane edges visible, overlaid with molecular weight markers and western blot bands from collagen gel samples with decreasing Col I proportions. Different exposure times (50 - 200 s) were used. Lane numbers correspond to the Col I proportions. A total of three individual membranes were analyzed, with membranes 1 and 2 used for quantifying blot intensity in Fig. 3B. (B) Full blot with arrows indicating the locations where the membranes were cut.

Supplementary Table

Table S1. Preparation of collagen gel matrices with various Col I/III ratios

Solution	1:0	9:1	4:1	7:3	3:2	0:1
Col I	0.666	0.6	0.534	0.466	0.4	0
Col III	0	0.2	0.4	0.6	0.8	1.268
Neutralization solution	0.038	0.046	0.054	0.06	0.068	0.072
10 × PBS	0.564	0.422	0.28	0.142	0	0

Unit : ml

Concentration : Col I ∼3 mg/ml; Col III ∼1 mg/ml; final solution ∼1.58 mg/ml (for pure Col III, the concentration is ∼ 1 mg/ml).

Final volume : 1.268 ml

Table S2. Summary of averaged mode values of χ_{15}/χ_{31}, χ_{33}/χ_{31} and θ^p for starch granules, human ECM, mouse ECM and mouse muscle. Values are presented as Mean ± SD, n= 5

parameter	starch granules	human ECM	mouse ECM	mouse muscle
χ_{15}/χ_{31}	0.26 ± 0.01	0.31 ± 0.02	0.38 ± 0.02	0.45 ± 0.01
χ_{33}/χ_{31}	1.55 ± 0.05	1.12 ± 0.02	1.14 ± 0.01	0.84 ± 0.01
θ^p	42.15° ±0.22°	46.36° ±0.07°	46.75° ±0.07°	49.81° ±0.59°

Table S3. Summary of averaged mode values of χ_{15}/χ_{31}, χ_{33}/χ_{31} and θ^p for collagen gel matrices. Values are presented as Mean ± SD, n= 10

Col I/III	1:0	9:1	4:1	7:3	3:2
χ_{15}/χ_{31}	0.51 ± 0.03	0.54 ± 0.03	0.55 ± 0.06	0.56 ± 0.06	0.55 ± 0.04
χ_{33}/χ_{31}	1.11 ± 0.01	1.12 ± 0.01	1.12 ± 0.03	1.12 ± 0.04	1.11 ± 0.02
θ^p	47.72° ±0.12°	47.85° ±0.12°	47.97° ±0.12°	48.04° ±0.15°	48.04° ±0.12°

Table S4. Summary of averaged mode values of χ_{15}/χ_{31}, χ_{33}/χ_{31} and θ^p for healthy (n = 5) and scar skin (n = 7). Values are presented as Mean ± SD.

parameter	healthy skin	scar skin
χ_{15}/χ_{31}	0.33 ± 0.03	0.34 ± 0.04
χ_{33}/χ_{31}	1.11 ± 0.02	1.12 ± 0.01
θ^p	46.73° ±0.15°	46.67° ±0.19°

Supplementary Video title and legend

Visulization 1. PSHG imaging from starch granule across 18 polarization angles. This video presents a PSHG image sequence of starch granule captured across 18 polarization angles. The dynamic visualization highlights the flow of maximum SHG intensity as the polarization angle changes. Regions of the granule become illuminated when the incident polarization direction aligns with the local orientation of the starch structure. White arrows indicate the current polarization angle applied in each frame.

Visulization 2. PSHG imaging from human extracellular matrix (ECM) across 18 polarization angles. This video showcases a PSHG image sequence of human ECM captured across 18 polarization angles. It demonstrates how SHG intensity varies as the polarization angle changes. Collagen fibers exhibit the highest intensity when the polarization direction aligns with their orientation. White arrows indicate the current polarization angle applied in each frame.

Visulization 3. PSHG imaging from mouse extracellular matrix (ECM) across 18 polarization angles. This video showcases a PSHG image sequence of human ECM captured across 18 polarization angles. It demonstrates how SHG intensity varies as the polarization angle changes. Collagen fibers exhibit the highest intensity when the polarization direction aligns with their orientation. White arrows indicate the current polarization angle applied in each frame.

Visulization 4. PSHG imaging from mouse mouscle across 18 polarization angles. This video showcases a PSHG image sequence of mouse muscle captured across 18 polarization angles. Myosin exhibit the highest intensity when the polarization direction aligns with their orientation. White arrows indicate the current polarization angle applied in each frame.
